# Attitude Toward Persons With Mental Illness in Bangladesh: Scale Validation and Status Assessment

**DOI:** 10.1002/puh2.70432

**Published:** 2026-10-29

**Authors:** S. M. Yasir Arafat, Sadeed Hossain, Md. Khairul Islam, Maliha Huq, Farhana Kaniz Mouri, Srijony Ahmed, Sanjida Tanjin Khan, Bithika Mali, Mohammad Sorowar Hossain

**Affiliations:** ^1^ Department of Psychiatry Bangladesh Specialized Hospital Dhaka Bangladesh; ^2^ Biomedical Research Foundation Dhaka Bangladesh; ^3^ Tairunnessa Memorial Medical College Gazipur Bangladesh; ^4^ National Institute of Preventive and Social Medicine Dhaka Bangladesh; ^5^ Department of Community Medicine and Public Health Medical College for Women and Hospital Dhaka Bangladesh; ^6^ Department of Psychiatry Dhaka Community Medical College and Hospital Dhaka Bangladesh; ^7^ Department of Psychiatry Ad‐din Medical College and Hospital Dhaka Bangladesh; ^8^ Department of Graduate Nursing Faculty of Nursing Bangladesh Medical University Dhaka Bangladesh; ^9^ School of Environment and Life Sciences Independent University Dhaka Bangladesh

**Keywords:** medical students, mental health, mental illness, psychometric validation, religious leaders

## Abstract

**Background:**

Stigma toward persons with mental illness remains a major barrier to mental health care. No validated Bangla instrument is available to assess attitudes toward persons with mental illness in Bangladesh.

**Objective:**

This study aimed to adapt and validate the culturally adapted Bangla version of the Attitudes Toward Persons with Mental Illness (APMI) scale and to assess attitudes among undergraduate medical students and Islamic religious leaders in Bangladesh.

**Methods:**

A psychometric validation study was conducted from July 2025 to June 2026 among 489 participants (249 medical students and 240 Imams). The original 16‐item APMI scale was translated and culturally adapted following standard guidelines. The sample was independently divided into exploratory factor analysis (EFA; *n* = 245) and confirmatory factor analysis (CFA; *n* = 244) subsamples. EFA used polychoric correlations, minimum residual extraction, oblimin rotation, and parallel analysis. CFA compared one‐ and two‐factor models using the WLSMV estimator. Reliability, measurement invariance, group differences, and multivariable associations were also evaluated.

**Results:**

EFA supported a two‐factor structure comprising Fear/Social Avoidance and Interpersonal Acceptance. The correlated two‐factor CFA model showed substantially better fit than the one‐factor model (scaled CFI = 0.967, TLI = 0.956, RMSEA = 0.071, 90% CI 0.049–0.092, SRMR = 0.046). Ordinal alpha was 0.83 and 0.78. Measurement invariance was supported at the configural, partial metric, and partial threshold/scalar levels. Medical students had lower scores than Imams. After adjustment, the difference remained significant (*β* = 5.02, 95% CI 2.89–7.15; *p* < 0.001).

**Conclusion:**

The 10‐item culturally adapted Bangla APMI scale demonstrates acceptable structural validity and internal consistency for assessing attitudes toward persons with mental illness for Bangla‐speaking populations.

## Graphical abstract

Stigma toward persons with mental illness remains a major barrier to mental health care. We validated Bangla attitudes toward persons with mental illness scale in Bangladesh. The 10‐item Bangla APMI scale is a reliable and valid instrument for assessing attitudes toward persons with mental illness.

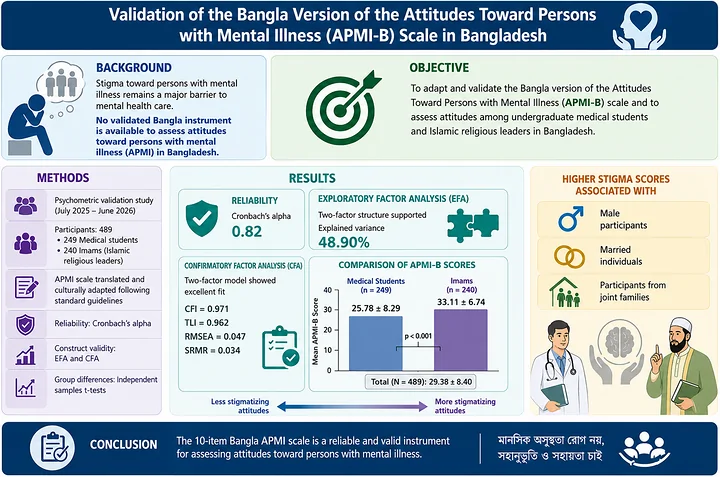

## 1 Introduction

Mental disorders are among the leading causes of non‐fatal disability worldwide, contributing substantially to the global burden of disease and affecting individuals across all age groups, cultures, and socioeconomic contexts [1]. Despite growing recognition of mental health as a public health priority, people living with mental illness continue to face pervasive stigma, discrimination, and social exclusion [2, 3]. Negative societal attitudes toward individuals with mental illness can adversely affect help‐seeking behavior, treatment adherence, social integration, employment opportunities, and overall quality of life [4]. Consequently, reducing stigma and promoting positive attitudes toward people with mental illness have become important components of global mental health strategies [5].

Bangladesh is a lower‐middle‐income country in South Asia with a population of more than 170 million, approximately 70% of whom reside in rural areas and more than 90% of whom are Muslim [6]. Mental disorders are prevalent across the population, with the National Mental Health Survey estimating that approximately 18.7% of Bangladeshis have a psychiatric disorder. Despite this burden, more than 90% of individuals with mental illness do not receive professional treatment [7].

While structural barriers such as a severe shortage of psychiatrists, a malfunctioning referral system, and a mental health budget of only around 0.5% of total health expenditure contribute significantly to this treatment gap, attitudinal and sociocultural factors represent equally important and often overlooked barriers [8]. Media portrayals of mental illness in Bangladesh frequently depict persons with mental disorders in negative, sensationalized, or misleading ways, which further reinforces public stigma, fear, and discriminatory attitudes [9]. As a result, persons with mental illness in Bangladesh commonly face social discrimination, difficulties in marriage, workplace discrimination, and shame within families [10].

Attitudes toward persons with mental illness represent an important but distinct construct from stigma. Stigma is a broader social process that may involve labeling, stereotyping, prejudice, status loss, discrimination, and reduced opportunities within power differentials [11], whereas attitudes refer to how individuals perceive, evaluate, and respond to those living with mental disorders. These attitudes encompass dimensions of social acceptance, willingness to interact, endorsement of rights, and attribution of responsibility for illness [12]. Negative APMI constitute a primary driver of social stigma, and both influence each other, which in turn discourages individuals with mental health problems from disclosing symptoms, seeking care, or remaining engaged in treatment [13]. Measuring and understanding these attitudes among influential population groups is therefore essential for designing effective anti‐stigma interventions and reducing the social exclusion of persons with mental illness in Bangladesh.

The APMI scale was originally developed to assess attitudes toward persons with mental illness [14]. However, direct application of an instrument developed in another linguistic and cultural context may compromise validity because the meaning and acceptability of individual items can vary across settings. Cultural adaptation and psychometric evaluation are therefore necessary before using the instrument in Bangla‐speaking populations.

The present study aimed to culturally adapt the APMI into Bangla and evaluate the psychometric properties of the resulting culturally adapted Bangla APMI‐B among medical students and Imams in Bangladesh. The findings are intended to inform anti‐stigma programming, medical education curricula, and community‐based mental health promotion strategies in Bangladesh.

## 2 Methods

### 2.1 Study Design and Settings

This psychometric validation study was conducted in Bangladesh between July 2025 and June 2026 among medical students and Imams in Bangladesh. Medical students were recruited from Tairunnessa Memorial Medical College, Medical College for Women and Hospital, Dhaka Community Medical College, while Imams were recruited from the Islamic Foundation, Bangladesh. Data from undergraduate medical students and Imams were collected using a self‐administered questionnaire.

### 2.2 Study Population

The study included two distinct participant groups: undergraduate medical students enrolled in recognized medical colleges in Bangladesh and active Islamic religious leaders currently serving in mosques or engaged in formal religious leadership activities.

These populations were selected due to their potential influence on societal attitudes toward individuals with mental illness. Medical students represent future healthcare professionals whose perceptions of mental illness are likely to affect clinical interactions, stigma reduction efforts, and future mental health service delivery.

Islamic religious leaders, particularly Imams, play a central role in shaping community beliefs and responses to mental illness in Bangladesh. With approximately 350,000 mosques nationwide [15], Imams are often among the first points of contact for individuals and families dealing with mental health‐related concerns. Evidence suggests that religious leaders frequently influence community interpretations of mental illness and decisions regarding help‐seeking and treatment pathways [16].

### 2.3 Eligibility Criteria

Participants were eligible for inclusion if they were aged 18 years or older, belonged to one of the two predefined study groups, were able to read and understand Bangla sufficiently to complete the self‐administered questionnaire independently, and provided written informed consent.

Participants were excluded if they were unable to complete the questionnaire independently because of cognitive, communication, or other functional difficulties at the time of data collection.

### 2.4 Sample Size

For psychometric validation, a participant‐to‐item ratio of at least 20:1 was adopted. Given the 16‐item adapted APMI scale, a minimum sample of 320 participants was targeted based on a conservative 20:1 participant‐to‐item ratio, ensuring adequate statistical power and stability of factor structures [17].

A total of 503 participants were initially recruited, including 259 medical students and 244 Islamic religious leaders. After exclusion of incomplete responses, the final analytical sample consisted of 489 participants (249 medical students and 240 Imams).

Since psychometric analyses benefit from independent samples for scale development and confirmation, the final sample was stratified by participant group and randomly divided into exploratory factor analysis (EFA) and confirmatory factor analysis (CFA) subsamples. The EFA subsample consisted of 245 participants (125 medical students and 120 Imams), while the independent CFA subsample consisted of 244 participants (124 medical students and 120 Imams).

### 2.5 Sampling Technique

A non‐probability purposive sampling technique was employed. We purposively selected medical students from medical colleges and Imams who were undergoing a training program arranged by the *Islamic Foundation, Bangladesh*.

### 2.6 Study Instrument

#### 2.6.1 Attitude toward Persons With Mental Illness Scale – Bangla Version

The APMI scale was adapted from the self‐report instrument used by Dawood and Modayfer [14], which was originally used to assess public attitudes toward mental illness and mental health services in Saudi Arabia. The original instrument consisted of 16 items. During the cultural adaptation process, items were reviewed, and those deemed culturally appropriate and relevant to the Bangladeshi context were retained, resulting in a refined 10‐item version used in the present study. For regular use, we aimed to pertain items related to regular daily life events. Therefore, during translation and back‐translation procedure, we dropped items related to rarely occurring life‐event related items, that is, “I can go in a picnic with people with mental illnesses,” “I refuse to marry a person with a family history of mental illness,” “I refuse to marry a person who had a mental illness and got treated,” “I visit mental health hospitals frequently,” and “I like to watch movies about mental illnesses” and one item which was not clear in language: “I don't refuse to set with a person with mental illness.” We ensured face validity and content validity during these phases consulting with expert committee review. Items 3, 5, 8, and 9 were reverse‐scored using the transformation (6—response), such that all items were oriented in the same direction. Each item is rated on a five‐point Likert scale, so after reverse‐scoring, the total APMI‐B scores were calculated by summing the 10 items, yielding a possible scores ranging from 10 to 50. Higher total scores indicate more unfavorable APMI.

The instrument does not have established diagnostic or severity cutoffs. Therefore, the total score was analyzed as a continuous measure and was not categorized into arbitrary levels of “low,” “moderate,” or “high” attitude.

#### 2.6.2 Translation and Cross‐Cultural Adaptation

The APMI scale was translated and culturally adapted into Bangla following internationally recognized guidelines for cross‐cultural adaptation of self‐report measures. The adaptation process aimed to ensure semantic, idiomatic, experiential, and conceptual equivalence between the original English version and the Bangla version [17].

The process involved five standard stages. First, two independent forward translations into Bangla were prepared by bilingual translators with different backgrounds, including one with a mental health background and one without clinical expertise allowing both conceptual and lay‐language perspectives to be considered. Second, the translations were compared and synthesized into a single preliminary Bangla version. Third, two independent who were blinded to the original instrument then performed back‐translations into English. Fourth, an expert panel consisting of three mental health professionals, two language experts, and four researchers reviewed all versions to ensure cultural relevance and conceptual equivalence. Fifth, the pre‐final version was pilot‐tested among 30 participants from the target population to assess clarity, comprehensibility, and cultural appropriateness, and minor linguistic refinements were made based on participant comprehension. Items that were judged to be insufficiently relevant or culturally applicable were subsequently considered for exclusion during the adaptation and validation process.

### 2.7 Data Collection and Quality Control

Data were collected using a structured questionnaire. Participants completed the questionnaire independently where feasible. Participation was voluntary, and written informed consent was obtained before data collection. Participants were informed about the purpose of the study and their right to decline participation or withdraw without penalty. No personally identifying information was included in the analytic dataset, and confidentiality was maintained during data handling and analysis. Completed questionnaires were reviewed immediately after collection for completeness and consistency. Forms with excessive missing data were excluded. Data were entered into a secured database using double‐entry procedures, and discrepancies were resolved by verification against original forms.

### 2.8 Statistical Analysis

Data were analyzed using IBM SPSS Statistics (version 22.0) and RStudio (version 4.5.1). Categorical variables were summarized using frequencies and percentages. Continuous variables were summarized using the mean and standard deviation (SD), median, interquartile range (IQR), and minimum–maximum range, as appropriate. The distribution of APMI‐B scores was examined using graphical methods, including histograms and boxplots, together with descriptive distributional summaries.

Internal consistency was assessed using Cronbach's alpha, ordinal alpha based on the polychoric correlation matrix, and McDonald's omega. Composite reliability (CR) and average variance extracted (AVE) were calculated for the CFA‐derived factors.

For EFA, the sample was randomly divided into EFA and CFA subsamples using stratified random sampling by participant group. EFA was conducted using a polychoric correlation matrix, minimum residual (minres) common‐factor extraction, and oblimin rotation. Sampling adequacy was assessed using the Kaiser–Meyer–Olkin (KMO) measure and Bartlett's test of sphericity. The number of factors was determined using parallel analysis, by the scree plot and consideration of the interpretability and theoretical coherence of the resulting factor solution. Factor retention was not based solely on the eigenvalue‐greater‐than‐one criterion. Items with their primary factor loadings and cross‐loadings were examined.

CFA was conducted in the independent CFA subsample using the weighted least squares mean and variance adjusted (WLSMV) estimator, with the 10 Likert‐type items specified as ordered categorical variables and theta parameterization. One‐factor and two‐factor models were evaluated. Model fit was assessed using the chi‐square statistic (*χ* ^2^), degrees of freedom (df), comparative fit index (CFI), Tucker–Lewis index (TLI), root mean square error of approximation (RMSEA) with its 90% confidence interval, and standardized root mean square residual (SRMR). Standardized factor loadings were examined to assess the relationship between items and their respective latent factors.

Measurement invariance of the two‐factor model across medical students and Imams was evaluated sequentially at the configural, metric, and threshold levels. Changes in CFI, TLI, RMSEA, and SRMR were considered when evaluating invariance.

Differences in APMI‐B scores between medical students and Imams were initially examined using independent‐samples *t*‐tests. Cohen's d and 95% confidence intervals were calculated to quantify the magnitude and precision of the between‐group difference. Secondary sociodemographic comparisons were treated as exploratory, and *p* values were adjusted using the Holm procedure to account for multiple testing.

Multivariable linear regression was used to examine the association between participant group and APMI‐B total score after adjustment for age, marital status, and family type. Sex was not included because it was perfectly confounded with participant group, as all Imams were male. Study year was unavailable for Imams and was therefore not included in the common multivariable model. Heteroscedasticity was assessed using the Breusch–Pagan test; because heteroscedasticity was detected, HC3 heteroscedasticity‐robust standard errors and 95% confidence intervals were used. Influential observations were assessed using Cook's distance, and a sensitivity analysis was performed excluding the observation with the largest Cook's distance.

All statistical tests were two‐sided, and *p* < 0.05 was considered statistically significant, except where adjusted *p* values were reported for exploratory multiple comparisons.

### 2.9 Ethical Considerations

The study protocol was reviewed and approved by the Ethical Review Board of the Biomedical Research Foundation, Dhaka, Bangladesh (Memo No. BRF/ERB/2025/002; dated March 15, 2025). All study procedures were conducted in accordance with the principles of the Declaration of Helsinki. Written informed consent was obtained from all participants prior to data collection. The confidentiality and anonymity of participants were strictly maintained throughout the study, and all data were used exclusively for research purposes.

## 3 Results

### 3.1 Sociodemographic Characteristics of Participants

A total of 489 participants were included in the study. The sample consisted of 310 males (63.4%), approximately half were undergraduate students (50.9%), and most participants were unmarried (55.8%) and predominantly Muslim (94.1%). A minority (14.1%) reported a history of mental illness, and 16.0% reported a family history of mental illness (Table 1).

**TABLE 1:** Sociodemographic characteristics of participants (*n* = 489).

Variable	Category	*n* (%)
Sex	Male	310 (63.4)
	Female	179 (36.6)
Education	Medical students	249 (50.9)
	Religious leaders	240 (49.1)
Marital status	Unmarried	273 (55.8)
	Married	208 (42.5)
	Divorced	2 (0.4)
	Widower	1 (0.2)
	Separated	5 (1.0)
Religion	Islam	460 (94.1)
	Hindu	27 (5.5)
	Buddhist	2 (0.4)
Family type	Nuclear family	320 (65.4)
	Joint family	164 (33.5)
	Missing	5 (1.0)
History of mental illness	Present	69 (14.1)
	Absent	419 (85.9)
Family history of mental illness	Yes	78 (16.0)
	No	411 (84.0)
Currently taking medication for mental illness	Yes	20 (4.1)
	No	468 (95.7)
Any medication taken for mental illness (lifetime)	Yes	52 (10.6)
	No	437 (89.4)
Family history of taking medication for mental illness	Yes	73 (14.9)
	No	416 (85.1)

The mean age of medical students was 23.55 ± 1.09 years (range 20–27), whereas the mean age of Imams was 32.34 ± 9.56 years (range 19–59). Eight participants had missing age data.

### 3.2 Validity Assessment

#### 3.2.1 Face and Content Validity

Face and content validity of the scale were assessed during the translation and cross‐cultural adaptation process through expert review of the translated items. The expert panel evaluated linguistic clarity, conceptual relevance, cultural appropriateness, comprehensibility, and conceptual equivalence between the original and Bangla versions of the scale. Items considered culturally inappropriate or less relevant to the Bangladeshi context were removed during the adaptation process. The questions enquiring about relatively infrequent social situations, such as going on a picnic, watching a movie together, marriage, and inviting a person with mental illness to social events, were excluded based on expert review and cultural relevance.

Face and content validity were assessed through expert review of the translated items, focusing on linguistic clarity, conceptual relevance, cultural appropriateness, and comprehensibility. Formal quantitative content‐validity indices, such as the item‐level content validity index (I‐CVI) and scale‐level content validity index (S‐CVI), were not calculated. This is acknowledged as a limitation of the present validation study.

### 3.3 Item Characteristics

The 10‐item and culturally adapted Bangla APMI‐B consisted of four reverse‐coded items (Q3, Q5, Q8, and Q9). Item means ranged from 2.81 to 3.09.

The independent EFA demonstrated a clear two‐factor structure. Factor 1, labelled Fear/Social Avoidance, consisted of six items (Q1, Q2, Q4, Q6, Q7, and Q10), whereas Factor 2, labelled Interpersonal Acceptance, consisted of four items (Q3, Q5, Q8, and Q9). Cross‐loadings were small, and no item required deletion based on the EFA solution.

### 3.4 Exploratory Factor Analysis

The EFA was performed using the independent subsample of 245 participants. A polychoric correlation matrix was used because the questionnaire items were ordinal. Minimum residual extraction with oblimin rotation identified two correlated factors.

Parallel analysis supported a two‐factor solution having eigenvalue >1 and the interpretability of the resulting factor structure. The first factor comprised six items (Q1, Q2, Q4, Q6, Q7, and Q10), with loadings ranging from 0.581 to 0.774. This factor was interpreted as Fear/Social Avoidance because its items represented fear, avoidance, and reluctance to engage socially with persons with mental illness.

The second factor comprised Q3, Q5, Q8, and Q9, with loadings ranging from 0.619 to 0.726. This factor was interpreted as Interpersonal Acceptance because the items concerned willingness to communicate, maintain friendship, and interact positively with persons with mental illness.

The two factors were moderately correlated (*r* = 0.549), supporting the use of an oblique oblimin rotation rather than orthogonal rotation. The two factors accounted for 46.7% of the common variance. No item showed a substantial cross‐loading or required deletion on the basis of the independent EFA.

### 3.5 Confirmatory Factor Analysis

The two‐factor structure identified in the EFA was evaluated in the independent CFA subsample of 244 participants. A competing one‐factor model was also estimated to assess whether the 10 items could adequately be represented by a single latent construct.

The one‐factor model demonstrated poor fit, with a scaled CFI of 0.825, TLI of 0.775, RMSEA of 0.160 (90% CI: 0.142–0.179), and SRMR of 0.093. In comparison, the correlated two‐factor model demonstrated substantially improved fit, with a scaled *χ* ^2^ of 75.45 (df = 34), CFI of 0.967, TLI of 0.956, RMSEA of 0.071 (90% CI: 0.049–0.092), and SRMR of 0.046. These findings provided support for the two‐factor structure over a unidimensional model.

Standardized factor loadings were statistically significant for all items and ranged from 0.529 to 0.787 for the Fear/Social Avoidance factor and from 0.650 to 0.718 for the Interpersonal Acceptance factor. The two latent factors were moderately correlated (*r* = 0.593), indicating that the dimensions were related but empirically distinguishable.

Distribution of item characteristics and factor loadings for the two‐factor model are also presented in Table 2, and the corresponding path diagram is shown in Figure 1.

**TABLE 2:** Distribution of item characteristics of APMI‐B.

Item	Item statement	Reverse‐coded	Mean ± SD	EFA: Fear/Social Avoidance	EFA: Interpersonal Acceptance	CFA standardized loading	Corrected Item‐Total Correlation
Q1	I feel afraid when I see any person with mental illness	No	2.91 ± 1.37	0.698	−0.14	0.726	0.48
Q2	I refuse to sit with a person with mental illness	No	2.86 ± 1.36	0.774	−0.01	0.743	0.58
Q3	I don't get upset if my neighbor is a person with mental illness	Yes	2.96 ± 1.42	−0.04	0.726	0.718	0.48
Q4	I don't visit people with mental illnesses at their homes	No	2.88 ± 1.37	0.658	0.17	0.586	0.55
Q5	I can speak with any person with mental illness	Yes	3.04 ± 1.38	0.05	0.707	0.708	0.51
Q6	I feel afraid to go out with any person with mental illness	No	3.01 ± 1.32	0.654	−0.05	0.529	0.42
Q7	I refuse to invite any person with mental illness to my home	No	2.81 ± 1.33	0.603	0.20	0.787	0.62
Q8	I can make friendship with a person with mental illness	Yes	2.91 ± 1.28	0.03	0.619	0.650	0.45
Q9	I don't feel afraid when dealing with persons with mental illnesses	Yes	3.09 ± 1.35	0.02	0.722	0.689	0.49
Q10	I don't listen to a speech of a person with mental illness	No	2.88 ± 1.35	0.581	−0.01	0.658	0.46

*Note:* EFA was conducted using minimum residual extraction with oblimin rotation in the independent EFA sample (*n* = 245). CFA was conducted in the independent CFA sample (*n* = 244) using WLSMV estimation. Higher total scores indicate more unfavorable attitudes.

**FIGURE 1:**
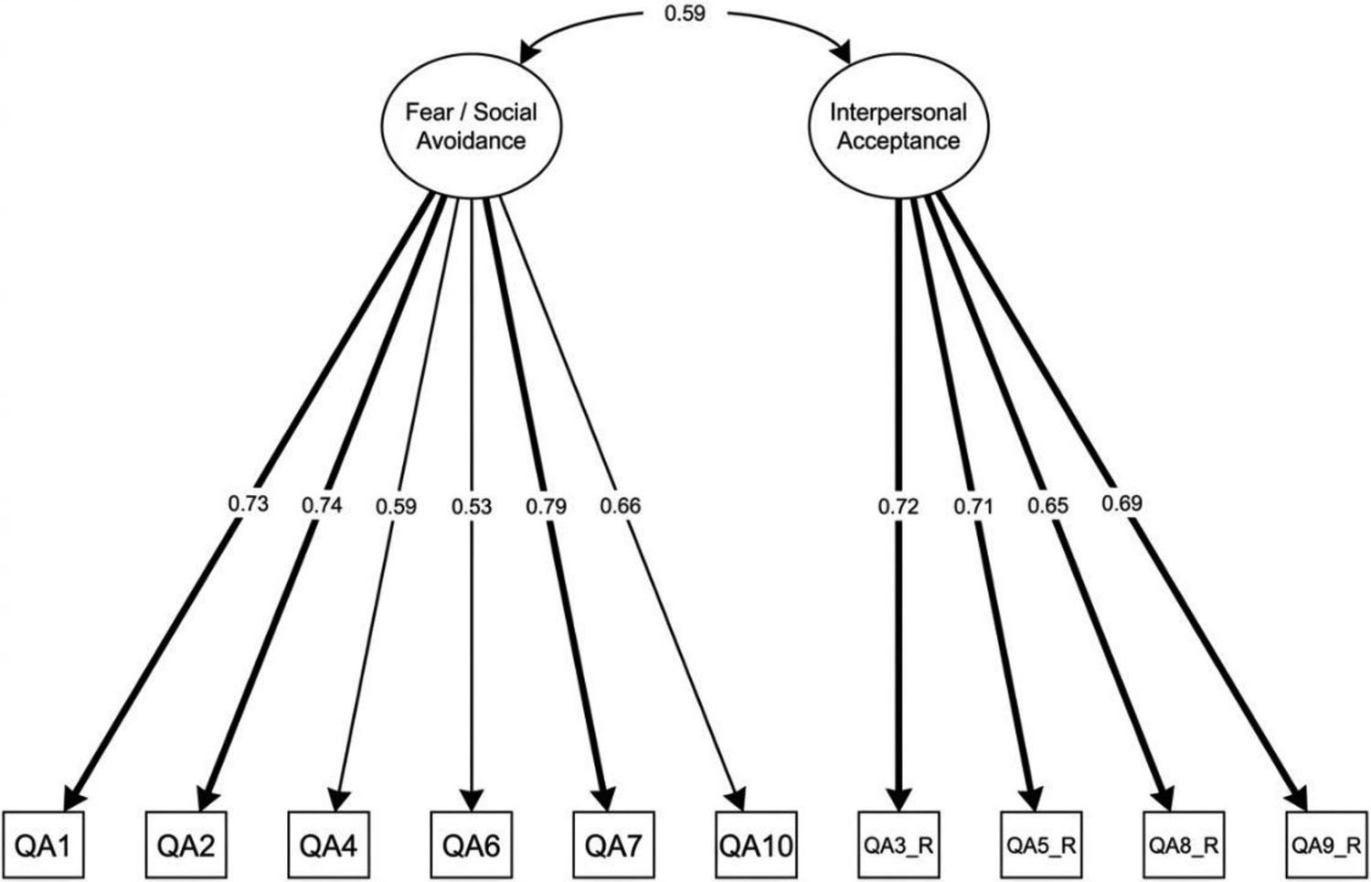
CFA path diagram of the APMI scale.

### 3.6 Reliability and Construct‐Related Evidence

Internal consistency was acceptable for both factors. Conventional Cronbach's alpha was 0.792 for Fear/Social Avoidance and 0.749 for Interpersonal Acceptance. Ordinal alpha was 0.825 and 0.784, respectively, while McDonald's omega was 0.828 and 0.785. Composite reliability was 0.833 for Fear/Social Avoidance and 0.786 for Interpersonal Acceptance.

The AVE values were 0.459 and 0.479, respectively, which are slightly below the conventional 0.50 threshold and therefore indicate borderline convergent validity. However, the CR estimates were acceptable, and all CFA loadings were statistically significant. The square roots of AVE were 0.678 and 0.692, respectively, both exceeding the inter‐factor correlation of 0.593, providing evidence supporting discriminant validity.

### 3.7 Measurement Invariance

Multigroup CFA was conducted to examine whether the two‐factor measurement structure operated similarly in medical students and Imams. The configural model showed CFI of 0.956, TLI of 0.942, RMSEA of 0.097, and SRMR of 0.071.

Full metric invariance was not supported, with CFI decreasing from 0.956 to 0.932 and RMSEA increasing from 0.097 to 0.114. Examination of group‐specific factor loadings identified a substantial difference for Q4, whose standardized loading was 0.816 among medical students and 0.247 among Imams.

After freeing the Q4 loading, the partial metric model demonstrated CFI of 0.950, TLI of 0.940, RMSEA of 0.098, and SRMR of 0.077. Compared with the configural model, the change in CFI was −0.006, and the change in RMSEA was +0.002. A subsequent partial threshold/scalar model retaining the freely estimated Q4 loading showed CFI of 0.944, TLI of 0.947, RMSEA of 0.092, and SRMR of 0.073. These findings supported configural, partial metric, and partial threshold/scalar invariance, but not full measurement invariance.

### 3.8 Differences in APMI Scores by Participant Group and Sociodemographic Characteristics

As shown in Table 3, the mean total APMI score for the overall sample was 29.38 (SD 8.40), suggesting attitudes that were generally centered around the midpoint of the scale. However, substantial variation was observed across participant subgroups.

**TABLE 3:** Comparison of APMI scores by participant group and sociodemographic characteristics.

Variable	*n* (%)	APMI	
		Mean (±SD)	*p* value
Total	489 (100)	29.38 (±8.40)	
Medical students	249 (50.9)	25.78 (±8.29)	**<0.001**
Imams	240 (49.1)	33.11 (±6.74)	
**Sex**			
Male	310 (63.4)	31.49 (±7.86)	**<0.001**
Female	179 (36.6)	25.69 (±8.06)	
**Marital status**			
Single	281 (57.5)	26.88 (±8.15)	**<0.001**
Married	208 (42.5)	32.75 (±7.54)	
**Religion**			
Islam	460 (94.1)	29.49 (±8.39)	0.196
Others	29 (5.9)	27.42 (±8.47)	
**Family type**			
Nuclear family	320 (66.1)	27.86 (±8.56)	**<0.001**
Joint family	164 (33.9)	32.44 (±7.23)	
**Mental Illness**			
Present	69 (14.1)	30.30 (±9.19)	0.329
Absent	419 (85.9)	29.24 (±8.28)	
**Family history of mental illness**			
Yes	78 (16.0)	30.47 (8.83)	0.208
No	411 (84.0)	29.17 (8.32)	
**Currently taking any medication for mental illness**			
Yes	20 (4.1)	32.55 (±10.17)	0.086
No	468 (95.9)	29.25 (±8.31)	
**Any medication taken for mental illness in lifetime**			
Yes	52 (10.6)	28.46 (±9.17)	0.408
No	437 (89.4)	29.48 (±8.32)	
**Family history of taking any medication for mental illness**			
Yes	73 (14.9)	28.67 (±9.39)	0.439
No	416 (85.1)	29.49 (±8.23)	

*Note: p* value was determined by independent *t* test. Significant *p* values are shown in bold.

Imams demonstrated significantly higher APMI scores than medical students, representing the largest observed group difference (33.11 ± 6.74 vs. 25.78 ± 8.29; mean difference = 7.33, 95% CI 5.99–8.68; Cohen's *d* = 0.97, 95% CI 0.78–1.16). Male participants, living in joint family, and married individuals had significantly higher scores (Table 3).

No statistically significant differences in APMI scores were observed according to religion, self‐reported history of mental illness, family history of mental illness, current use of psychiatric medication, lifetime use of psychiatric medication, or family history of psychiatric medication use (Table 3).

### 3.9 Multivariable Regression

Missing data were examined at the variable and item levels before analysis (Table 4). Age was missing for eight participants, while the final 10 APMI‐B items had no missing responses. The CFA was conducted in the prespecified CFA subsample (*n* = 244). The multivariable regression used complete cases for the variables included in the model, resulting in an analytic sample of 476 participants.

**TABLE 4:** Multivariable linear regression of APMI‐B total score.

Predictor	β	HC3 SE	95% CI	*p* value
Intercept	23.18	1.40	20.44 to 25.92	<0.001
Imam vs. medical student	5.02	1.08	2.89 to 7.15	<0.001
Age, per year	0.10	0.06	−0.01 to 0.21	0.080
Unmarried vs. married	1.37	1.15	−0.89 to 3.63	0.234
Joint vs. nuclear family	1.20	0.83	−0.44 to 2.84	0.152

*Note:* Reference categories were medical students, married, and nuclear family. The model included complete cases (*n* = 476). HC3, heteroscedasticity‐consistent standard errors. Sex was not included because it was perfectly confounded with participant group. Study year was unavailable for Imams.

In the unadjusted linear regression model, Imams had APMI‐B scores 7.33 points higher than medical students (95% CI 5.99–8.68; p < 0.001).

The multivariable model was adjusted for age, marital status, and family type. The Breusch–Pagan test indicated heteroscedasticity (BP = 13.01, df = 4, *p* = 0.011); therefore, HC3 heteroscedasticity‐robust standard errors were used.

As shown in Table 4, following adjustment, Imams had APMI‐B scores that were, on average, 5.02 points higher than those of medical students (HC3 SE = 1.08; 95% CI 2.89–7.15; *p* < 0.001). Age was not significantly associated with APMI‐B score (*β* = 0.10 per year; 95% CI −0.01 to 0.21; *p* = 0.080). Neither unmarried status (*β* = 1.37; 95% CI −0.89 to 3.63; *p* = 0.234) nor joint family structure (*β* = 1.20; 95% CI −0.44 to 2.84; p = 0.152) was statistically associated with APMI‐B score. The adjusted model explained approximately 20% of the variance in APMI‐B scores (adjusted *R* ^2^ = 0.198).

A sensitivity analysis excluding the observation with the largest Cook's distance produced a similar group estimate (*β* = 5.25), supporting the robustness of the association.

## 4 Discussion

### 4.1 Major Findings

The present study represents a cross‐cultural adaptation and psychometric validation of the culturally adapted Bangla version of the APMI‐B among undergraduate medical students and Islamic religious leaders in the context of Bangladesh, a Muslim‐majority, lower‐middle‐income country.

EFA using polychoric correlations, minimum residual extraction, and oblimin rotation identified two correlated dimensions: Fear/Social Avoidance and Interpersonal Acceptance. These factors were subsequently supported in an independent CFA sample, and the two‐factor model showed substantially better fit than the one‐factor model. Standardized factor loadings were satisfactory across all items.

Internal consistency was acceptable across both factors, with ordinal alpha values of 0.83 and 0.78 and omega coefficients of 0.83 and 0.78 for Fear/Social Avoidance and Interpersonal Acceptance, respectively. Composite reliability was also acceptable. However, AVE values were slightly below the conventional 0.50 threshold (0.459 and 0.479), suggesting that evidence for convergent validity remains somewhat borderline. Thus, the findings support the structural validity and internal consistency of the scale but do not establish all forms of validity.

Measurement invariance analysis further showed configural invariance, partial metric invariance, and partial threshold/scalar invariance between medical students and Imams. Full metric invariance was not supported, largely because of the markedly different loading of Q4 between the two groups. Therefore, comparisons between the groups should be interpreted with some caution rather than as evidence of complete measurement equivalence.

Medical students demonstrated lower APMI scores compared to Imams, indicating that medical students held more positive or less stigmatizing attitudes toward persons with mental illness than their Imam counterparts. The finding of relatively less stigmatizing attitudes among medical students is consistent with other global studies [18]. However, because the study was cross‐sectional and the groups were not randomly assigned, the findings represent an association and should not be interpreted as evidence that medical education itself reduces unfavorable attitudes.

Previous research from Bangladesh has shown that medical students may develop more positive views toward psychiatry during later years of medical education while simultaneously showing increased stigma toward people with mental disorders [19]. Therefore, the lower scores observed among medical students in the present study should not be interpreted as evidence of a sustained reduction in unfavorable attitudes throughout medical training. Longitudinal studies following students across different stages of training would be required to examine such a developmental pattern.

The study concluded that improvements in attitudes toward psychiatry were outweighed by the increase in stigma, showing the need for specific anti‐stigma training in the medical curriculum. This suggests that the lower APMI‐B scores found among medical students in the current study may not reflect a lasting reduction in stigma. Instead, stigma could increase again as students move into later clinical training if there are no targeted anti‐stigma interventions.

The higher APMI‐B scores among Imams suggest greater stigmatizing APMI. However, this cannot be simply explained by their beliefs about the causes of mental illness. Other studies found that many Bangladeshi Imams supported scientific explanations such as hereditary factors, brain chemical imbalance, stressful life events, and childhood trauma, while supernatural causes such as demonic possession received the lowest support and viewed medication positively [16, 20].

Despite these science‐based beliefs, Imams still showed more stigmatizing APMI in the present study. This suggests that having accurate knowledge about mental illness does not automatically reduce stigma. Negative attitudes, such as fear, social distance, and beliefs about dangerousness, may continue even when people accept scientific explanations [21, 22].

A UK based study found lower stigma among Muslim faith leaders who frequently interacted with people experiencing mental illness and regularly referred them to mental health services [23]. This indicates that direct personal contact may play a stronger role in reducing stigma than knowledge alone. Also educational interventions using active case studies and problem‐based learning can reduce stigma toward mental disorders [24].

Male participants demonstrated significantly higher APMI scores than female participants, indicating comparatively greater stigmatizing attitudes among males. This finding is consistent with previous studies from Bangladesh and other regions reporting higher levels of stigma toward mental illness among men [25, 26]. However, the interpretation of sex‐related differences requires particular caution because all Imams were male, whereas the medical‐student group included both male and female participants. Consequently, sex was perfectly confounded with participant group and could not be included as an independent covariate in the overall regression model. This prevents the present study from determining how much of the observed group difference may be attributable to sex rather than participant role. In the present study, age was not independently associated with APMI‐B score after adjustment. Similarly, the previously observed differences according to marital status and family type should be interpreted cautiously. In the multivariable model, neither marital status nor family type was statistically associated with APMI‐B score after adjustment for the other covariates. Therefore, these variables should not be presented as independent predictors of unfavorable attitudes.

Participants from joint families demonstrated significantly higher stigmatized attitudes than those from nuclear families, and married participants scored higher than unmarried participants, both indicating greater stigmatizing attitudes. These findings diverge from a strand of South Asian evidence that has associated joint family living and marriage with social support, shared caregiving, and lower psychiatric morbidity [27, 28].

### 4.2 Implications for Research, Policy, and Practice

This study has important implications across research, policy, and clinical practice. The shortened 10‐item and culturally adapted Bangla APMI‐B provides preliminary evidence of a two‐dimensional structure comprising Fear/Social Avoidance and Interpersonal Acceptance and may be useful for research involving Bangla‐speaking populations similar to those represented in this study. Future longitudinal studies are especially needed to examine how stigma changes during medical training, as Giasuddin et al. [19] found that stigma toward people with mental illness increases in later years of medical education despite improved attitudes toward psychiatry.

The finding that Imams showed science‐based beliefs about mental illness but still had higher stigma scores also needs further study [16, 20]. Future mixed‐methods research should examine which aspects of stigma such as fear, social restriction, or desire for distance are most influential in this group and whether contact‐based interventions work better than knowledge‐based approaches. Future studies should also test measurement invariance of the APMI‐B across different professional groups before making further comparisons.

From a policy perspective, the findings highlight the need for targeted anti‐stigma strategies in both medical education and community leadership settings. The observed differences between medical students and religious leaders suggest that stigma‐reduction policies should not focus solely on the general population but also on key influential groups who shape public opinion. The comparatively higher stigmatizing attitudes among Imams suggest the need for targeted interventions. The Islamic Foundation Bangladesh and the Imam Training Academy could incorporate mental health literacy into formal Imam training programs, building on the positive biomedical beliefs reported by Arafat et al. [16]. Such initiatives represent a scalable and culturally appropriate approach for reducing community‐level stigma and improving referral of persons with mental illness to appropriate care.

In terms of practice, integrating structured mental health literacy and anti‐stigma training into medical curricula may help improve attitudes among future healthcare professionals [29]. Similarly, engaging religious leaders in mental health awareness programs may strengthen community‐based referral pathways and improve early help‐seeking behavior, given their influential role in community support systems [30].

Future research should employ longitudinal designs to examine whether targeted educational interventions can shift attitudes in both groups and should explore the relationship between attitudes, help‐seeking behavior, and referral patterns in the Bangladeshi community context.

### 4.3 Strengths and Limitations

This study has several strengths. To our knowledge, this study validate and provides psychometric evidence for a shortened and culturally adapted Bangla version of the APMI scale in a Bangla‐speaking population. The inclusion of two socially and culturally distinct groups, that is, medical students and Islamic religious leaders, allowed meaningful comparative insight into attitudes toward mental illness in Bangladesh.

Several methodological strengths should also be noted. First, the study used an independent EFA/CFA design, reducing the risk of capitalizing on sample‐specific factor structures. Second, the EFA used a common‐factor method with an oblique rotation and polychoric correlations appropriate for ordinal responses. Third, factor retention was determined using parallel analysis, supplemented by visual inspection of the scree plot and consideration of factor interpretability, rather than relying solely on the eigenvalue‐greater‐than‐one criterion. Fourth, the CFA explicitly compared one‐ and two‐factor models. Fifth, measurement invariance was examined across the two participant groups. Finally, the analysis incorporated multivariable regression, effect sizes, confidence intervals, robust standard errors, and sensitivity analysis.

Notwithstanding these strengths, several limitations must be acknowledged. First, the use of non‐probability purposive sampling may have introduced selection bias and limits the generalizability of the findings to medical students and Islamic religious leaders beyond the participating settings. The study was also conducted within selected institutional contexts, and the findings may not necessarily extend to broader community, occupational, geographic, or cultural populations in Bangladesh.

Second, the cross‐sectional design precludes causal inference and does not allow the assessment of changes in attitudes over time. The observed differences between medical students and Imams may reflect unmeasured differences in age, sex, educational background, social exposure, socioeconomic circumstances, or other characteristics rather than participant group alone. In particular, sex was perfectly confounded with participant group because all Imams were male, preventing independent adjustment for sex in the overall regression model. Study year was also unavailable for Imams and therefore could not be incorporated as a potentially relevant covariate.

Third, measurement invariance between the two participant groups was only partially supported. Although configural, partial metric, and partial threshold/scalar invariance were supported, Q4 demonstrated a substantial difference in factor loading between medical students and Imams. Therefore, complete measurement equivalence cannot be assumed, and between‐group comparisons should be interpreted with appropriate caution.

Fourth, although the two‐factor model demonstrated acceptable internal consistency and structural validity, some aspects of construct validity remain to be established. The AVE values were slightly below the conventional 0.50 threshold, indicating borderline convergent validity. Moreover, convergent validity against established measures and criterion‐related validity were not assessed. Test–reliability was also not evaluated; therefore, the temporal stability of the shortened APMI‐B remains unknown. Inter‐rater reliability was not assessed because the instrument is a self‐administered self‐report measure and does not involve independent raters.

Fifth, the use of self‐reported responses may have introduced social desirability bias, particularly because attitudes toward persons with mental illness are socially sensitive. Although confidentiality and anonymity were emphasized, respondents may still have been inclined to report attitudes perceived as more socially acceptable.

Sixth, although face and content validity were considered during expert review as part of the translation and cultural adaptation process, formal quantitative content‐validity indices, such as the item‐level content validity index (I‐CVI) and scale‐level content validity index (S‐CVI), were not calculated. Consequently, the content validity of the shortened Bangla APMI‐B could not be quantified using standardized indices.

Finally, the final Bangla APMI‐B is a shortened, culturally adapted version of the original 16‐item instrument. Although the removal of culturally less applicable items may improve contextual relevance and measurement efficiency, item deletion may also reduce the breadth of construct coverage. The shortened instrument should therefore not be assumed to capture every aspect represented by the original scale. Further validation in larger and more diverse Bangla‐speaking samples, including community and occupational populations across different regions of Bangladesh, is warranted to evaluate the scale's temporal stability, convergent and criterion‐related validity, content coverage, and measurement equivalence across broader populations.

## 5 Conclusion

The 10‐item culturally adapted Bangla APMI‐B demonstrated promising structural validity and acceptable internal consistency, with evidence supporting a correlated two‐factor structure comprising Fear/Social Avoidance and Interpersonal Acceptance. The two‐factor model showed substantially better fit than a unidimensional model, while measurement invariance analyses supported configural, partial metric, and partial threshold/scalar invariance across medical students and Imams. Medical students demonstrated more favorable attitudes toward persons with mental illness than Imams, and this difference remained significant after adjustment for age, marital status, and family type.

The findings support the use of the Bangla APMI‐B as a research measure of attitudes toward persons with mental illness in populations similar to those studied, rather than as a diagnostic or clinical classification instrument. Further studies involving larger and more diverse Bangla‐speaking populations should evaluate test–retest reliability, convergent and criterion‐related validity, content coverage, and measurement equivalence across additional demographic and occupational groups.

## Author Contributions

**S. M. Yasir Arafat**: conception. **Md. Khairul Islam, Farhana Kaniz Mouri, Srijony Ahmed, Sanjida Tanjin Khan**, and **Bithika Mali**: data collection. **S. M. Yasir Arafat, Sadeed Hossain, Maliha Huq, Farhana Kaniz Mouri, Srijony Ahmed, Sanjida Tanjin Khan**, and **Bithika Mali**: data curation and analysis. **Mohammad Sorowar Hossain**: supervision. **S. M. Yasir Arafat** and **Sadeed Hossain**: writing – original draft. **S. M. Yasir Arafat, Md. Khairul Islam, Sadeed Hossain, Maliha Huq, Farhana Kaniz Mouri, Srijony Ahmed, Sanjida Tanjin Khan, Bithika Mali**, and **Mohammad Sorowar Hossain**: writing – review and editing. All authors have read and approved the final version of the manuscript.

## Funding

No funding was received for this paper.

## Ethics Statement

The study protocol was reviewed and approved by the Ethical Review Board of the Biomedical Research Foundation, Dhaka, Bangladesh (Memo No. BRF/ERB/2025/002; dated March 15, 2025). All study procedures were conducted in accordance with the principles of the Declaration of Helsinki. Written informed consent was obtained from all participants prior to data collection. Confidentiality and anonymity of participants were strictly maintained throughout the study, and all data were used exclusively for research purposes.

## Conflicts of Interest

The authors declare no conflicts of interest.

## Data Availability

The data that support the findings of this study are available from the corresponding author upon reasonable request.
